# Constipation in children

**DOI:** 10.1186/1824-7288-37-28

**Published:** 2011-06-13

**Authors:** Nadeem A Afzal, Mark P Tighe, Mike A Thomson

**Affiliations:** 1 Paediatric Department, Southampton University Hospitals NHS Trust, Southampton General Hospital, Tremona Rd, Southampton, Hants, SO16 6YD, England; 2 Paediatric Department, Poole Hospital NHS Trust, Longfleet Rd, Poole, BH15 2JB, England; 3 Department of Paediatric Gastroenterology, The Sheffield Children's NHS Trust, Western Bank, Sheffield, S10 2TH, England

## Abstract

Constipation remains a frequent presentation to paediatricians, with significant health resource implications. We present a practical guide to the management of paediatric constipation and evaluate the current evidence for treatment regimens, to help the clinician in treating a condition that can be distressing and has a significant impact on affected families.

## Introduction

Constipation is a very common presentation, both in primary and secondary care. Prevalence of functional constipation in children ranges from 4-36% [1-3]. In the hospital setting, paediatric constipation forms 3% of all referrals to paediatric practice and up to 25% to paediatric gastroenterologists. In addition a recent American study suggests that there is a cost of health resources for children with constipation, estimated at $3.9 billion/year [4].

This article aims to be a practical guide for paediatricians and primary care physicians, to outline the current diagnostic criteria and provide an evidence-base for the medical management of idiopathic constipation in children, in the light of recent National Institute of Clinical Excellence (NICE) guidelines on constipation [5]. With increased patient and parental understanding and support, as well as improving toileting habit, increasing fibre and optimising laxatives; the potential exists to deliver a significant benefit to children.

## Normal Bowel Habit in Children

Considerable variation in 'normal' bowel habit in children is accepted. In a UK based study of 350 pre-school children (1-4 years of age), 96% of the children passed bowel motions between 3 times a day to alternate daily [6]. Stool frequency is also age-dependent. Nyhan, in a study of 800 babies described a peak frequency of 4.4 per day at 5 days of age [7], and may be as high as 13 per day in breast fed infants. Although a difference in stool frequency may exist between breast and bottle fed babies, time to first stool after passage of meconium remains the same in both groups [8].

## Definition

The wide variation in normal defaecation patterns in children discussed above makes it difficult to define constipation. Normal ranges also vary with age and place of residence [9].

Functional constipation has been defined by the ROME III classification [10] as 2 or more of the following features in a child with a developmental age of at least 4 years and occurring at least once per week for at least 2 months before diagnosis (with insufficient criteria for diagnosis of irritable bowel syndrome).

• 2 or fewer defaecations in the toilet per week

• At least 1 episode of faecal incontinence per week

• History of retentive posturing or excessive volitional stool retention

• History of painful or hard bowel movements

• Presence of a large faecal mass in the rectum

• History of large diameter stools that may obstruct the toilet

More recently, the term 'non-retentive faecal soiling' has been described for children soiling without difficult infrequent defaecation. PACCT (The Paris Consensus on Childhood Constipation Terminology Group) have defined this as passage of stools in an inappropriate place, occurring in children with a mental age of 4 years and older, with no evidence of constipation on history or examination [11]. The Iowa criteria of constipation in children ≥ 2 years of age include two or more of the following during the previous 8 weeks: 

≥1 episodes of faecal incontinence per week

Large stools in the rectum or felt on abdominal examination

Passing of stools so large that they obstruct the toilet

Retentive posturing (withholding behaviour)

Painful defecation

< 3 bowel movements per week.

How sensitive and specific are these definitions? In a recent Turkish study on 485 children being treated for constipation, 33 children (6.8%) were not recognised by the Rome III criteria used in the study, due to problems with age restriction. 45 (9.2%) children were not recognized using the PACCT criteria due to only having scybalous, pebble-like defaection pattern (rather than passing large stools obstructing the toilet). Only 60% had a defaecation pattern of less than 3 per week [12].

Given below are details of individual symptoms and their significance in relation to the definitions above.

## Symptoms of constipation

### 1. Infrequent stools

Reduced bowel movement is commonly used to make a diagnosis. In a study of 178 children with constipation in Iowa, 58% had < 3 bowel movements per week [9] and in another study 41.3% of children with symptoms of constipation were found to have infrequent stools [13]. Children < 2 years of age had constipation with symptoms of passage of hard or pebble-like stools with straining, withholding or painful defaecation. The diagnosis would be missed in 50% if infrequent stools were the only criteria used for diagnosis.

### 2. Pain

Children may present with pain in the abdomen or during defaecation. Non-specific abdominal pain has been reported in 33% of children with constipation in one study. Painful defaecation occurs when children complain of pain or scream during or on anticipation of stools, observed in up to 68% of children with constipation [9]. They may pass blood with stools (see below).

### 3. Soiling

Faecal incontinence has been associated with 'constipation' in up to 90% [13]. Soiling is involuntary, often small and stains the underwear, however if larger in amount can be mistaken for diarrhoea. Medications may be incorrectly reduced which may instead need to be maintained or increased.

### 4. Stool withholding manoeuvres

This may be misinterpreted as straining. In infants back-arching, and in older infants/toddlers, standing on toes, extending legs or rocking back and forth preventing anal relaxation are typical features. Some children may hide in a corner standing stiffly or squatting.

### 5. Blood in stools

Fissures may result in bleeding and painful defaecation in older children. Children may present with blood on tissue paper after wiping. Perineal examination should include looking for infection/cellulitis, fissures, fistulae or tags. The latter, associated with faltering growth or delayed puberty, may be suggestive of Crohn's disease. Comparatively children with polyps commonly present with painless bleeding. Rectal bleeding in infancy is often associated with cow's milk protein allergy rather than constipation [14].

### 6. Enuresis and other urinary symptoms

Urinary symptoms have been reported in 9-13% of children with a diagnosis of constipation, and urinary incontinence 10.5%, and it has been implicated in the patho-aetiology of enuresis [13]. Asymptomatic constipation may exacerbate urinary symptoms in children with enuresis [15]. The impacted stool in the rectum compresses the bladder, reduces its functional capacity, and provokes earlier sensation to void. In addition, chronic pelvic floor spasm prevents complete relaxation during voiding, and contributes to postvoid residuals [16-18].

### 7. Associations

1. Obesity [19,20]

Overall a higher incidence of obesity has been found in constipated children, compared to the general paediatric population, with associated psychosocial issues, poor diet, low activity levels, and compliance problems.

2. Poor Fluid intake

Optimal fluid intake is recommended by NICE as a necessary adjunct in the management of constipation [5]. However, excessive fluid intake can result in reduction in eating/fibre, which may be counter-productive [21].

## Epidemiology

A positive family history has been found in 28-50% of constipated children and a higher incidence reported in monozygotic than dizygotic twins [22]. Constipation tends to be equal in both sexes below 5 years, commoner in girls above 13 years of age, and peak incidence is at the time of toilet training around 2-3 years of age [23].

## Aetiology of idiopathic constipation

Understanding the trigger for constipation in children is important. This may occur secondary to inadequate evacuation as a result of rushing to school in the morning, quick use of the school toilet, the child withholding stools as they may be occupied by something of greater interest. Occasionally children may have had a hard stool due to decreased fluid intake after a febrile illness or during a holiday trip.

Children with difficult toilet training are more likely to be constipated. These children may be less adaptable and negative in mood. 74% would hide stool and 37% would ask for pull ups to leave the stool in it [24]. These children benefit more from constant encouragement using star charts/other reward techniques, rather than confrontation (see below).

Secondary constipation, for instance due to hypothyroidism, Hirschsprung's disease, or changes in calcium levels is rare and accounts for less than 10% of cases. However cow's milk protein allergy, particularly non-IgE mediated, with associated colonic dysmotility may manifest as secondary constipation [25,26], with one study estimating its prevalence in up to 40% of refractory constipation [27].

Up to 63% of children with constipation and faecal soiling will have a history of painful defaecation beginning before 3 years of age and secondary withholding behaviour [28,29]. Stool with-holding follows passing a hard painful bowel motion, creating a vicious cycle of pain leading to further withholding, stool hardening and increase in size with subsequent megarectum, lack of defaecatory signal etc. Parents may mistake such withholding behaviour as straining.

## Diagnosis

Constipation is diagnosed by clinical history and examination. History should include a detailed exploration of symptoms, looking at potential precipitants, and for 'red flags' to exclude organic pathology (see table 1). A physical examination should include an abdominal examination to assess the degree of faecal loading, as well as neurological assessment of the spine and lower limbs. Perineal examination helps to look for perianal cellulitis and anorectal anomalies. There is now clear NICE guidance on rectal examinations in children that these should only be performed by healthcare professionals who are competent in recognising anorectal anatomical problems and Hirschsprung's disease, and this examination can provide useful information on sphincter tone, and rectal loading. Constipation should be considered as a differential diagnosis in all children presenting with abdominal pain [30]. In a child with an underlying neurological diagnosis or developmental delay, irritability may be suggestive; and a high index of suspicion maintained, as constipation may be present in the context of gut dysmotility. Constipation may be missed in 1/3^rd ^of children with autism if clinical criteria alone are used to make a diagnosis [31]. Other considerations include an association between children with refractory constipation and abuse but features such as soiling should be placed into context and are not discriminatory in isolation [32].

**Table 1:** Red Flags: Common symptoms and signs to suggest organic causes of constipation

History	Examination
**Failure to thrive**	**Absent/Brisk lower limb reflexes**

**Delayed passage of meconium**	**Mouth ulcers**

**Abnormal bowel habit since birth**	**Blood/mucus mixed in with stool**

**Sensitivity to cold, fatigue, dry skin, pallor**	**Perianal skin tags or fistulae**

**Change of bowels with introduction of cow's milk**	**Associated hypotonia**

**Weight loss**	**Fever**

It is important to watch for growth failure, 'overflow diarrhoea' with blood and/or mucus, pallor or fatigue, or failure to respond to conventional treatment during the course of management, and be prepared to re-evaluate. Children with inflammatory bowel conditions (with or without anal involvement) and coeliac disease may present with constipation. It is important to remember that like fever, constipation may be a symptom and not a diagnosis.

Finally, even in idiopathic constipation, it is useful to understand the underlying mechanics and family dynamics, which, if not addressed, may often lead to failure of intensive laxative treatment.

## Investigations

Often tests are not needed and only conducted to exclude secondary constipation. A nutritional assessment may be part of the initial blood screen which includes screening for thyroid and coeliac disease [5]. In children with constipation specific IgE to cow's milk is not diagnostic of cow's milk allergy [25;26].

### Plain abdominal X ray

Constipation is a clinical and not a radiological diagnosis. Occasionally a plain abdominal × ray is useful in cases of diagnostic uncertainty, but remains highly subjective. Scoring systems have been trialled to improve consistency. One system, the Leech system [33] divides the abdominal × ray into three sections, ascending colon and proximal transverse colon; distal transverse colon; descending colon and rectosigmoid area. Each segment is then assessed for the presence of stools (score 0-5 where 0 indicates no stool; 5 means gross faecal loading with bowel dilation). The Leech system has been shown to be superior to two other systems (Barr and Blethyn) by two paediatric radiologists for validity (kappa values of 0.88 and 1.00, P < 0.05) [34]. In another study X-rays were comparatively scored by a student, junior doctor and consultant. The results suggested that scoring by these systems is dependent on the experience of the observer, and does not accurately discriminate between constipated children and children without constipation [35].

Presence of firm, packed hard stool in the rectum correlates closely with radiological evidence of faecal retention, with sensitivity and positive predictive values exceeding 90% [33,36,37].

A 'Shapes' bowel transit study, where a patient ingests three different radio-opaque marker shapes on consecutive days and a subsequent × ray on day 4 identifies where these markers are, can be very helpful in determining specific anatomical area of hold up. For instance recto-sigmoid accretion of markers would be observed in long-standing constipation of idiopathic origin, such as with stool withholding, whereas markers may be distributed more widely throughout a dysmotile colon.

### Ultrasound

Bijoś has described using ultrasound (USS) for diagnosis of constipation [38]. The transverse diameter of the rectal ampulla increases with age and thus influenced the USS measurements in both the patient and control groups. The numerical values of this parameter differed significantly between patients and controls in all age groups. The rectopelvic ratio is the ratio of the width of the rectal ampulla (on USS) to the distance between the anterior superior iliac spines (measured externally using a measuring tape) and has been used to define 'megarectum'.

### Anorectal/Colonic Manometry

This is not a first line investigation but has been used in children with refractory symptoms, often with failed multiple treatments, who may have had required multiple hospitalisations for treatment of their symptoms. Presence and normal propagation of high amplitude propagating contractions (HAPCs) with presence of a gastro-colonic response is suggestive of intact neuromuscular function. The highest prevalence of motor abnormalities have been reported in children with intestinal pseudo-obstruction [39]. Colonic manometry can be useful for planning surgical intervention in children with refractory symptoms.

### Rectal biopsy

Deep suction rectal biopsy is the gold standard for diagnosing Hirschsprung's disease. If the age at onset of constipation is after the neonatal period, Hirschsprung's disease is very unlikely [40]. Ultra short Hirschsprung's disease is rare, and was first described by Davidson and Bauer in 1958 [41]. Strip biopsies are recommended to avoid falsely negative biopsies. Strip biopsies include mucosa from the dentate line to the rectum, and a full thickness biopsy is preferable.

## Management

The practicing physician should be up to date with the principles of management. Recently published data from Virginia (USA) suggested that up to 86% of the primary care physicians had no awareness of the published clinical guidelines for constipation in children [42]. After 2 months of treatment, nearly 40% of children remained symptomatic; which was improved by improved parental understanding and regular laxatives [43].

### Education

Parental/family education regarding their understanding of the aetiology, symptoms and principles of management remain critical in achieving success. Management starts with explaining the physiological basis of constipation and soiling to the child and family. NICE recommend that the child should never be blamed for soiling and this should be explained to parents [5].

The family should be encouraged to adhere to the treatment plan (both medication and a regular toileting pattern) with emphasis on its efficacy for long-term symptom improvement. Underlying psychosocial problems should be considered in the first meeting. These may range from bullying to pressure to use a single family toilet quickly. Clear and simple messages should be given to avoid overwhelming the family.

Toileting at school may need to be addressed with the teacher with involvement of the school nurse. Boyt [44] in a postal questionnaire in Iowa, described lack of awareness of most teachers regarding childhood constipation. One third of respondents indicated that they ask children to wait to go to the bathroom. Suboptimal conditions existed in most school toilets, with only 35% of the boys' toilets and 48% of the girls' toilets reported as "always clean".

### Diet

NICE recommend optimising fibre intake [5,45-47]. Children with normal defaecation patterns in comparison to children with constipation have better fibre intake. A strong family history of cow's milk protein intolerance, or raised eosinophil count, and elevated specific IgE to cow's milk, would merit a trial of cow's milk protein-free diet, and in infants, a hydrolysed formula [26]. A trial of cow's milk protein-free diet is also merited for non-IgE-mediated cow's milk protein intolerance, in those with a suggestive history, for example if constipation started on switching from breast- to formula-feeding [48], or in those with refractory constipation [25]. Increased fluid intake is recommended [5;49], but not at the expense of excessively restricting calorie intake in younger children.

## Evidence base for medications used in treatment of constipation in children

### Search criteria

**Figure 1:**
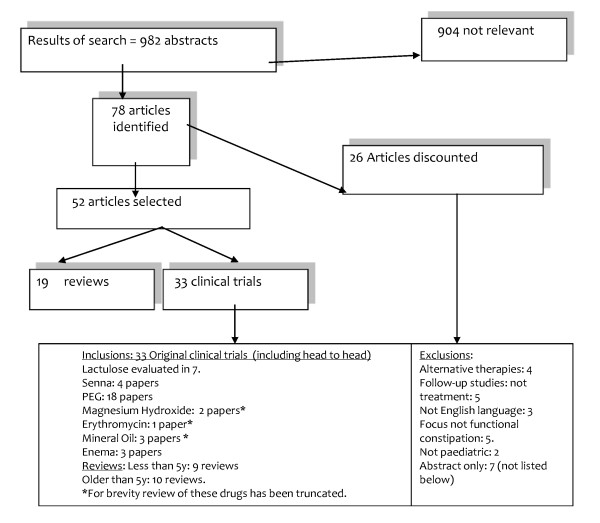
Flow Chart

We searched PubMed, Medline, and Embase and then hand search reviews from the past 5 years for 'constipation', 'soiling', 'faecal', 'fecal', 'incontinence', 'child$', 'infant', 'baby', 'drugs', 'therapy' and 'treatment'. Reviews and abstracts are excluded. We appraised the original clinical trials using the Levels of Evidence adopted by the Oxford Centre for Evidence-based Medicine, and produced evidence-based recommendations [50]. We have constructed a flow chart, showing the outcome of the search, the number of excluded papers by reason for exclusion and number of papers selected for review (Figure 1).

### Disimpaction

Disimpaction is important, particularly in severe cases, for maintenance treatment to work, according to NICE [5]. An escalating dose of polyethylene Glycol (PEG) is recommended as first-line treatment (see evidence below) with a stimulant added if required. The family should be prepared for potential worsening of overflow soiling initially. An excessive dose of stimulant risks precipitating acute abdominal pain in cases of impaction. If possible, such treatments should commence during weekends/holidays to minimise stress for the child and family. Diarrhoea in a constipated child initialising a treatment is likely to represent spurious overflow diarrhoea and the treatment would need to be increased or at least maintained rather than reduced. It is sensible to review the child regularly. In practical terms, this may mean 2-3 appointments 1-2 weeks apart initially. Specialist nurses can play a major role during this period. Disimpaction should generally be initiated orally; further discussion of enemas is outlined below.

### Maintenance therapy

Once disimpaction has taken place, the aim of laxative treatment, as recommended by NICE, should be to keep the child, symptom free with regular soft bowel actions and should be commenced immediately [5], initially at half the dose required for disimpaction. PEG is the first line treatment, with a stimulant laxative such as senna added if required, or substituted if PEG is not tolerated. During this period, the child should be encouraged to use the toilet regularly, particularly 15-30 minutes after meals. A useful rule of thumb is to aim for 1-2 soft stools a day, though this is not definitive. Laxative treatment needs to be tailored to the child; and toilet training can often be as important as the choice of laxatives. Give parents the support to allow them to vary the laxative dose in response to their child's symptoms (for example through telephone advice from a specialist nurse). Once improvement is seen, laxatives should be gradually reduced and never stopped suddenly. Improvement in chronic cases may take place over months and can take years in some cases. Reinforcement of the management plan may be required. Relapses are common and may need to be treated with increasing doses of laxatives. A reward system with star chart is often used with success. Finally, toilet training should be postponed in a constipated 2-year old until after disimpaction.

Choice of laxatives may vary between regions and countries. Lactulose, macrogols, and Senna (table 2, 3, 4) are popularly used treatments in the UK. In the section below we have looked at the available evidence of efficacy of these medications, with an abbreviated discussion of mineral oil, erythromycin and magnesium hydroxide (due to the paucity of evidence). The evidence base for enemas (table 5) is also reviewed.

**Table 2:** Lactulose

Authors	Study Group+ type	Methods + Key Outcomes
Voskuijl et al (2004) [51]	100 patients Study type :1b	Methods: 8 wk double blinded, multi-centre RCT: PEG 3350 vs lactulose. Results: Success greater for PEG group (56%) vs. lactulose group (29%) (less pain/straining). PEG significantly less palatable

Van Ginkel (2000) [52]	48 children Study type :2b	Methods: RCT: Biofeedback+ lactulose vs. biofeedback for 7 weeksResults: Both groups had improved encopresis (group 2 significantly better (86%) than group 1 (53%) p < 0.01).

Gremse et al (2002) [59]	44 children Study type :1b	Methods: Unblinded Crossover RCT Lactulose vs PEG 3350: 2 weeks Results: Lactulose had a significantly longer mean transit time compared to PEG 3350 (55.3 vs. 47.6 hrs, p = 0.038). Stool form, frequency, and ease of passage were similar for each laxative.

Perkin (1977) [53]	21 children Study type :2b	Methods: Randomised unblinded crossover study lactulose vs senna:3 weeks Results: Lactulose more likely to give greater number of days when normal stools were passed (p < 0.01). Side-effects significantly higher (p < 0.001) for senna.

Dupont et al (2005) [54]	96 children Study type :2b	Method: Random allocation, open label cohort study Results: More than 90% of children recovered normal bowel habits. Faecal mass in the rectum and abdominal pain were markedly reduced and appetite improved.

Keuzen-kamp et al (1996) [55]	244 patients Study type :4	Methods: Case series over 25y: median follow up of 4 years 3 phases: 1) evacuation with lactulose/bisacodyl 2)maintenance: regular toileting + wean laxatives to ensure soft stools daily 3) introduce high fibre diet +wean laxatives. Results: 66% cured, 34% still symptomatic, 12 had persistent symptoms. Of all 39% had at least one recurrence.

Connolly et al (1974) [56]	164 patients Study type :4	Methods: Open-label cross-over study: lactulose vs. irritant laxatives: (Senna/bisacodyl) 7 days Results: 58% of lactulose group vs 42% of stimulant group had normal stool.

**Table 3:** Macrogols (Polyethylene Glycol)

PEG 4000		
**Authors**	**Study Group** **Study type**	**Methods+Key Outcomes**

Thomson et al (2007) [57]	51 children Study type : 1b	Methods: Double-blind crossover RCT PEG+E or placebo for 2 weeks Results: Mean number of defaecations higher for PEG+E group vs placebo (p < 0.001). Also PEG+E reduced pain on defaecation (p = 0.041), straining on defaecation (p < 0.001), stool consistency (p < 0.001) and percentage of hard stools (p = 0.001). Adverse events were all mild or moderate and were similar for those children on PEG+E and placebo.

Candy et al (2006) [58]	63 children Study type : 2b	Methods: Initial open cohort study of PEG+E (disimpaction) then double-blind RCT of PEG+E (Movicol) vs. lactulose (maintenance) Results: Disimpaction) successful in 92% children. Maximum dose = 4 sachets -4 yr old) or 6 sachets (5-11 yr olds); median time to disimpaction was 6 days. Maintenance: Greater mean stool frequency in PEG + E group (p = 0.007).

Dupont et al (2005) [54] - Study described in the 'Lactulose section' - table		

**PEG 3350**		

Gremse et al (2002) [59] Study described in the 'Lactulose section' - table		

Loening-Baucke et al (2006) [60]	79 children Study type : 2b	Methods: Double blind RCT PEG 3350 vs. magnesium hydroxide Results: Significant improvement in both groups, (frequency of bowel movements, reduced frequency of incontinence, and resolution of abdominal pain). Compliance = 95% (PEG) vs. 65% = milk of magnesia. At 12 months, 62% of PEG-treated children and 43% of MoM-treated children improving:.

Youssef et al (2002) [61]	40 children Study type : 1b	Methods: Prospective double-blind, parallel RCT 4 doses of PEG 3350 Results: Disimpaction in 75% of children overall but significant difference between two higher doses vs. lower doses (95% vs. 55%, P < .005). All groups had an increased number of bowel movements during the 5-day study versus baseline.

Michail et al (2004) [62]	28 patients	Method: Cohort study with PEG. Mean duration 6 months Results: Mean effective maintenance dose was 0.78 g/kg/day. PEG relieved constipation in 97.6% of patients.

Voskuijl et al (2004) [51] Study described in the 'Lactulose section' - table		

Ingebo (1988) [63]	24 patients Study type : 4	Methods: Case series: PEG+E given at 14-40ml/kg/hr until clear fluid obtained. Results: children with encopresis required an average of 11L over 22 h. PEG+E successful in all children, and was safe.

Miller et al (2007) [64]	121 patients Study type : 4	Methods: Case series: single-site: All children over 6 month period diagnosed with 'constipation' from an Emergency Department. Results: 2/3 had had pain for less than 1 week. 70% received an AXR. 1/3 received an enema in the ED. 74% received laxatives on discharge (80% given PEG). At follow-up, 35% were using laxatives, and 27% had sought additional care. After an enema, 28% discharged without laxatives.

Pashankar et al (2003) [66]	83 children Study type : 2b	Methods: Cohort study for at least 3 m PEG given at 0.8 g/kg/day then adjusted to give 2 soft painless stools/day Results: Mean duration = 8.7 months. Mean PEG dose was 0.75 g/kg daily. No major adverse effects. All children preferred PEG to other laxatives, Good daily compliance in 90% of children

Pashankar et al (2003) [65]	74 children Study type :4	Methods: Case series: All given PEG 3350 for > 3 months. Results: mean duration of PEG therapy = 8.4 m (3-30 m). Weekly stool frequency, stool consistency, and soiling improved significantly with PEG therapy in all patients.

Pashankar et al (2001) [67]	24 children Study type : 4	Cohort study: PEG for 8 weeks starting at 1 g/kg/d: adjusted every 3 days aiming: 2 soft stools/d. Results: Weekly stool frequency increased from 2.3 to 16.9 (p < .0001) with treatment. Stool consistency improved (p < .0001). Mean effective dose was 0.84 g/kg/d (range, 0.27-1.42 g/kg/d)

Erickson (2003) [68]	46 patients Study type : 4	Method: Retrospective review of PEG 3350, aiming for 2 soft stools a day. Results: PEG caused significant increase in frequency of bowel movements (p = 0.0001). Average final dose was 0.63 gm/kg. Diarrhoea in 9 patients. 18 became dry, 26 had decreased wetting (2 no improvement).

Hanson (2006) [69]	23 children Study type : 4	Method: Case series: 7 day disimpaction then maintenance PEG. Results: 23 questioned on their children's experiences. 96% 'more than happy' with PEG+E. Prior to PEG+E treatment, 57 per cent of children were admitted to hospital and 26 per cent required home visits for constipation treatment. After treatment, no child visited hospital/needed home visit.

Loening-Baucke et al (2004) [70]	75 constipated children < 2 years Study type : 4	Method: Retrospective case series: PEG. Results: Mean effective short-term PEG dose was 1.1 g/kg/day and the mean long-term dose was 0.8 g/kg/day. Constipation was relieved in 85% with short-term and in 91% with long-term PEG therapy.

**Table 4:** Senna

Authors	Study Group+ type	Methods+ Key Outcomes
Berg (1983) [71]	44 children with soiling (mean age 7.9 years) Study type 2b All given toileting advice.	RCT: senna vs. placebo vs. no treatment. Results: All groups improved from baseline (p < 0.05). Senna no more effective than placebo/no treatment.

Perkin (1977) [53]- Study described in the 'Lactulose section' - table		

Sondheimer et al (1982) [72]	37 children Study type: 2b	Methods: Non-blinded RCT Senna vs. mineral oil for 3 months Results: Faecal soiling + decreased stool frequency) significantly better in mineral-oil group. At least 1 recurrence of symptoms occurred in 66% of mineral-oil-treated and 89% of Senna-treated patients.

Connolly et al (1974) [56] -Study described in the 'Lactulose section' - table		

**Table 5:** Enemas

Authors	Study Group Study type	Methods+ Key Outcomes
Loening-Baucke (1993) [9]	174 children Study type: 4	Methods: case series: Long-term follow-up questionnaire. All given education, enemas for disimpaction, and dietary fibre+ magnesium hydroxide. Results: On presentation 64% impacted +received enema. 57/90 (63%) had recovered. 17 (19%) still required laxatives, and 16 (18%) still soiling regularly.

Borowitz et al (2005) [43]	119 children Study type: 4	Methods: Case series: Follow-up: Colonic evacuation then magnesium hydroxide (77%), senna syrup (23%), mineral oil (8%), and lactulose (8%) Results: Children who underwent some form of colonic evacuation followed by daily laxative therapy were more likely to have responded to treatment (p < 0.05).

Miller et al (2007) [64]	See PEG section above	

### Lactulose (see table 2) [51-56]

#### Summary

Older case series of variable quality point to some benefit. However RCTs have shown either inferiority to PEG (Voskuijl et al, Dupont et al) or absence of benefit (von Ginkel, Gremse et al).

#### Conclusions

Evidence points to a lack of benefit from lactulose, but more studies are needed (Grade B).

### Polyethylene Glycol (see table 3) [51,54,57-70]

#### Summary

Certainly PEG is now recommended as first line treatment as per UK NICE guidelines [5]. The studies cover the different scenarios of treatment: oral resolution of impaction and maintenance therapy for relief of constipation. Both PEG +E and PEG 3350 were evaluated. The largest studies (Thomson *et al*, Candy *et al*, Dupont *et al*, Youssef *et al*) demonstrate efficacy in resolution of symptoms from chronic constipation. Candy *et al *(2^nd ^phase of study) found that PEG gave greater stool frequency and had less adverse effects than lactulose. Michail *et al *evaluated the dose range for children < 18 m and found that a safe and effective dose was 0.78 g/kg/day. Loening-Baucke *et al *and Pashankar *et al *found a similar dose was safe and effective for older children over a mean period of over 8 months.

#### Conclusions

PEG is safe and effective in the treatment of impaction and chronic constipation. (grade B)

### Senna: (see table 4) [53,56,71,72]

#### Summary

4 low-quality studies provide little evidence of benefit from Senna. Sondheimer et al found that mineral oil (compared to senna) was more likely to reduce the frequency of soiling and recurrence of symptoms of constipation. Perkin found that lactulose was significantly more likely to give greater number of days of normal stool motions compared to senna. Berg compared senna to placebo and found no significant differences in the number of soiling episodes per week. UK NICE guidance recommends stimulant use second line to PEG solutions [5].

#### Conclusions

Based on current evidence there is no benefit seen from senna, although no high-quality trials exist to date. (Grade D)

### Enemas: (see table 5) [9,43,56]

#### Summary

Enemas in these case series were used to relieve impaction prior to initiation of maintenance therapy. The authors would recommend a RCT comparing enema vs. no enema relief of faecal impaction to further assess the impact enemas have, both in the acute management and in the medium to long-term.

#### Conclusion

More evidence is needed to assess the role of enemas in impaction. (Grade D).

#### Erythromycin

1 study of 14 children showed some significant results: Further evaluation through RCTs is needed to assess erythromycin as an adjunct in chronic constipation [73].

#### Magnesium hydroxide

1 paper showed magnesium hydroxide inferior to PEG [60]. More evidence is needed to evaluate the role of magnesium hydroxide in constipation (grade D)

#### Mineral Oil

3 papers were identified. Gleghorn et al [74] showed that mineral oil was effective in treating impaction in children, and Tolia [75] showed that whilst mineral oil may be better tolerated, PEG may be more effective. Sondheimer in a non-blinded study showed mineral oil to be superior to Senna in reducing constipation recurrences when used over 3 months [72]. More evidence is needed to assess the role of mineral oil in treating constipation (Grade D).

## Management of difficult/refractory constipation

Initial history and examination should be undertaken at the first appointment to exclude an organic aetiology and active investigations (as mentioned before) should be undertaken. If these reveal evidence of slow colonic transit, this may suggest a neuronal disorder of the intestine, which may require colonic manometry and a full thickness biopsy for definitive diagnosis.

As discussed earlier, continued extraneous factors such as bullying, adverse family dynamics and child abuse should be considered. Equally, children with neurological/psychiatric conditions may find it difficult to learn/adhere to a toileting routine. Negotiated and non-punitive behavioural interventions in conjunction with medicinal and dietary treatment is recommended by NICE guidance [5]. A period of inpatient admission may be useful. This can be aided by a multi-disciplinary team, including nurses experienced in managing children with constipation. Psychologists and play therapists can introduce methods of relaxation for toileting and dieticians can also input into fluid intake and fibre content.

Surgery should only be considered for appropriate organic indications or if medical management at a tertiary level fails, and is beyond the scope of this article.

## Prognosis

A high proportion of relapses have been reported after success in the initial treatment. These relapses have been reported to be commoner in boys than girls [76]. However the prognosis for constipation in under fives is excellent, with constipation resolving in 88% of children in this age group, when followed over an eighteen-month period. The non-responders came from families with increased degree of psychosocial problems where reduced compliance of medications was suspected [77]. In general 50% of children with chronic constipation will be cured after a year and 65-70% after 2 years, with much higher rates in motivated, adherent families [78]. Two studies show 34-37% to be still constipated 3-12 years after start of treatment [9,55].

## Conclusion

In this article we have outlined the current trends in the assessment and treatment of constipation and reviewed the current evidence-base for the therapies currently in wide use, within the context of recent NICE guidance. Constipation remains a prevalent problem, which can have a huge impact on children's quality of life, and places a burden on primary and secondary care. With increased patient and parental understanding and support, as well as improving toileting habit, increasing fibre and optimising laxatives; the potential exists to deliver a significant benefit to children, and revolutionise what can otherwise be an intractable and distressing condition.

## Competing interests

The authors declare that they have no competing interests.

## Authors' contributions

MAT and NAA conceived the article. MPT performed and wrote the literature search, and NAA wrote the article. MPT and NAA edited the article for publication. All authors have read and approved the script
